# Supplementary material for: A set of multi-entry identification keys to African frugivorous flies (Diptera, Tephritidae)
Source: Zookeys. 2014 Jul 24;(428):97–108. doi: 10.3897/zookeys.428.7366 (PMC4143993; doi:10.3897/zookeys.428.7366)
Supplement: Supplementary material 4 — Key to Capparimyia [file zookeys-428-097-s004.zip › SF4_ZooKeys_key to Capparimyia/lucid_player/help/help/the_lucid_players_menu_and_button_bar.htm]

The Lucid Player�s Menu and Button Bar

 


# The Lucid Player�s Menu and Button Bar

#### The Key menu

Restart  
Open  
Subsets  
Keep Subsets on Restart  
Allow Misinterpretations  
Retain Uncertains  
Matching Method (*Any State*, *All States*)  
Exit

#### The Features menu

Find Best/Sort Best  
Next Best  
Previous Best  
Best Mode (*Find Best*, *Sort Best*)  
Prune Redundants  
Restore Redundants  
Automate (*Auto Best*, *Auto Prune*)  
Shortcuts  
Display (*Image Gallery*)

#### The Entities menu

Sorting Mode
(*Filtered, Ranked*)  
Differences  
Why Discarded  
Display (*Image Thumbnails*)

#### The View menu

Language  
Font  
Font Size (*Smallest* to *Largest*)  
Refresh Item Trees

#### The Window menu

Options to set focus to an already open window, such as a fact sheet.

#### The Help menu

Help  
About Key  
About Lucid

#### The Button Bar
